# [Retracted] PLGA/poloxamer nanoparticles loaded with EPAS1 siRNA for the treatment of pancreatic cancer *in vitro* and *in vivo*

**DOI:** 10.3892/ijmm.2024.5459

**Published:** 2024-11-18

**Authors:** Xinting Pan, Qingyun Zhu, Yunbo Sun, Liandi Li, Yunpeng Zhu, Zhihui Zhao, Jianxin Zuo, Wei Fang, Kun Li

Int J Mol Med 35: 995-1002, 2015; DOI: 10.3892/ijmm.2015.2096

Following the publication of this article, a concerned reader drew to the Editor's attention that, in the tumor tissue images shown in Fig. 5A and B on p. 1001, there were two pairs of overlapping data panels, such that data which were intended to show the results from differently performed experiments appeared to have been derived from the same original sources. Subsequently, after having conducted a separate investigation in the Editorial Office, it came to light that there were also matching data panels intending to show the results from different experiments in the flow cytometric plots shown in Fig. 2; moreover, there appeared to be potential issues with the presentation of some of the western blots in Fig. 3.

Although the possibility of a corrigendum was considered, upon reflection, the Editor of *International Journal of Molecular Medicine* has decided that, owing to the number of problems that were identified with the data in the published paper, the article should be retracted from the publication on account of data mishandling issues and an overall lack of confidence in the presented data. The authors were asked for an explanation to account for these concerns, but the Editorial Office did not receive a reply. The Editor apologizes to the readership for any inconvenience caused, and thanks the interested reader for initially drawing this matter to our attention.

